# Estimating Cetacean Carrying Capacity Based on Spacing Behaviour

**DOI:** 10.1371/journal.pone.0051347

**Published:** 2012-12-07

**Authors:** Janelle E. Braithwaite, Jessica J. Meeuwig, K. Curt S. Jenner

**Affiliations:** 1 The Centre for Marine Futures (UWA Oceans Institute) and School of Animal Biology, Faculty of Natural and Agricultural Sciences, The University of Western Australia, Perth, Western Australia, Australia; 2 Centre for Whale Research, Fremantle, Western Australia, Australia; University of Western Ontario, Canada

## Abstract

Conservation of large ocean wildlife requires an understanding of how they use space. In Western Australia, the humpback whale (*Megaptera novaeangliae*) population is growing at a minimum rate of 10% per year. An important consideration for conservation based management in space-limited environments, such as coastal resting areas, is the potential expansion in area use by humpback whales if the carrying capacity of existing areas is exceeded. Here we determined the theoretical carrying capacity of a known humpback resting area based on the spacing behaviour of pods, where a resting area is defined as a sheltered embayment along the coast. Two separate approaches were taken to estimate this distance. The first used the median nearest neighbour distance between pods in relatively dense areas, giving a spacing distance of 2.16 km (±0.94). The second estimated the spacing distance as the radius at which 50% of the population included no other pods, and was calculated as 1.93 km (range: 1.62–2.50 km). Using these values, the maximum number of pods able to fit into the resting area was 698 and 872 pods, respectively. Given an average observed pod size of 1.7 whales, this equates to a carrying capacity estimate of between 1187 and 1482 whales at any given point in time. This study demonstrates that whale pods do maintain a distance from each other, which may determine the number of animals that can occupy aggregation areas where space is limited. This requirement for space has implications when considering boundaries for protected areas or competition for space with the fishing and resources sectors.

## Introduction

An important consideration for conservation is the population size that a given habitat can support. Estimating this carrying capacity provides a baseline against which changes to habitat can be assessed with respect to the maintenance of conservation values [1]. Here, carrying capacity is defined in terms of density limitation in a particular area at a given time, rather than the overall population carrying capacity (K) [2]. The limit to animal density in an area is generally related to the total amount of resources available in the habitat and the resource needs of each individual. It is well recognized that density scales inversely with body size across many plant and animal communities [3]–[6], as does home-range size in top predators [6]–[8]. Individual energy demand is the main explanation for these trends, with larger animals requiring more food and thus a larger area for foraging. Therefore, carrying capacity is often calculated based on food supply [9], [10]: for example, the estimated carrying capacity of sites used by migratory birds is calculated using a ‘daily ration model’, whereby the total consumable food of the site is divided by the individual energetic requirement [1], [10], [11]. However, this conventional approach to calculating carrying capacity is limited, and other studies have found that carrying capacity can also be influenced by predation risk [12], freshwater availability [13], shelter [14], and the availability of nesting sites [15]. As the space requirement of an animal, for example its home range, is generally related to the availability of resources, space itself can be considered as a resource that will limit density.

According to Tilman [16] “all things consumed by a species are potentially limiting resources for it”, where the term ‘consumed’ describes those things used, such as an occupied wood hole for a squirrel. Following this definition, we argue that space is a resource, as animals consume space due to the physical requirements to perform behaviours, such as individual fish within a school [17], or due to a behavioural preference of the animal, for example social density in primates [18]. The concept of space as a resource is also reflected in research into the welfare needs of animals in captivity, such as livestock or zoo animals with welfare positively correlated to size and complexity of enclosures. A classic example is caged hens (*Gallus gallus domesticus*), where a behavioural study on the confinements of laying hens in the late 1980s found that the existing cage measurements, based on the physical size of the bird (excluding wing-span), did not permit essential behaviour movements for the hens [19], [20]. Increased space availability in livestock has shown to improve welfare, such as playfulness in juveniles [21], conflict avoidance [22], [23], and reduced muscle damage and fatigue during transportation [24], [25]. In aquaculture, the stocking density of fish can affect growth rate [26] and mortality [27], however this is not only associated with the behavioural requirement of space for the individual, but with having space to allow for the circulation of high quality water and flow rates [27]. A study by Clubb and Mason [28] claims that success for carnivores in captivity is linked to home-range sizes in the wild, whereby infant mortality and stereotypic locomotive behaviour was positively correlated with increasing natural home-range sizes. In captivity food is plentiful, suggesting that the space use and natural ranging behaviour of carnivores in the wild can be a factor when considering animal welfare in captivity, regardless of the correlation between home-range size and foraging needs. Many of these examples are of animals in captivity and there has been little research on space as a resource in wild populations. Yet in naturally confined environments, the space requirements of an individual will determine the density limitation of animals in that area.

Migrating humpback whales in resting areas present a unique opportunity to investigate spacing behaviour in the wild, and the potential limitation this may have on the carrying capacity of the area. During migration, adult humpback whales are not actively feeding, eliminating energy requirements as a factor in density limitation. While calves and juveniles are feeding to varying degrees (Jenner, pers. obs.), their typical presence within a pod containing a fasting adult, where calves are feeding on their mother’s milk, means that it is unlikely to be a contributing factor to density limitation. Resting areas are found in relatively enclosed coastal areas, which provide shelter from open oceanographic conditions and protection from potential predators such as killer whales (*Orcinus orca*), and are therefore space limited. Along the coast of Western Australia, the use of coastal areas by the migrating humpback population is an important conservation issue; the humpback whale population is increasing at near maximum rates [29], while the coastline is becoming increasingly developed. For example, the large offshore oil and gas developments around the Pilbara region of Western Australia have resulted in the creation and expansion of coastal ports, increases in marine vessel traffic and noise, potentially creating competition with migrating whales for space in the ocean. This competition for space is of particular concern in resting areas, which provide the distinct conditions for humpback whales to rest, but are also limited in available space.

Here, we used innovative techniques to explore the concept of a space-defined carrying capacity in a natural environment by examining the spacing behaviour of humpback whales in Exmouth Gulf, a recognized resting and nursing area [30], [31], during the 2004 and 2005 migrations. Temporal use was estimated using aerial line-transect surveys, and overall space use was investigated through the abundance-occupancy relationship. Two different approaches were then used to determine the average distance maintained between pods. This spacing distance was calculated across whale pods in various behavioural states, to obtain a representative distance across the population occupying the Gulf at that point in time. Based on this space use we determine the carrying capacity of the area, which represents the theoretical maximum number of whales able to occupy Exmouth Gulf during the 2004–2005 seasons. We highlight the implications of having a space-defined carrying capacity in the context of an expanding population given current temporal and spatial use of the Gulf.

## Materials and Methods

### Study Area

**Figure 1:**
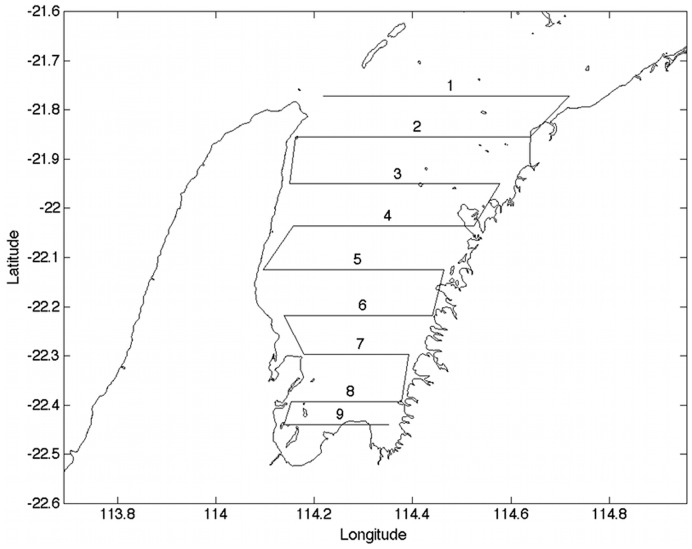
The aerial survey track over Exmouth Gulf. A typical course flown by the aircraft during surveys. This flight path was split into nine parallel transects spaced approximately 10 km apart.

Exmouth Gulf (Fig. 1) is located on the Northwest shelf of Australia, between 21°45’S–22°33’S and 114°08’E–114°40’E. This embayment is approximately 3000 km^2^ in size, with a mean depth of 9 m and maximum depth of about 20 m. The Gulf is located in the tropical zone and experiences an average SST of 22–23°C during October when whale numbers peak. Exmouth Gulf is a recognized resting area for breeding stock D humpback whales as they migrate southwards from their calving grounds in Camden Sound (northern Western Australia) to the Southern Ocean each year between August and November [31]. The Gulf is constrained by coastline on three sides, with a northern opening to the ocean.

### Aerial Surveys

A total of 17 aerial surveys were conducted in Exmouth Gulf between 7^th^ July 2004 and 15^th^ October 2005, of which 10 flights included observations of humpback whales (Table 1). Surveys were conducted in a twin-engine, overhead winged aircraft (Cessna 337) maintaining a cruising speed of 222 kmh^−1^ (120 knots) and an altitude of 305 m (1000 feet). Data were collected using distance-sampling methods, with the plane following a systematic parallel line transect course across the Gulf (Fig. 1) in passing mode (no deviations from the track), following Buckland et al. [32]. The parallel transects were spaced approximately 10 km apart to minimize overlap in the covered strips [29], [33]. Personnel aboard the aircraft included the pilot, two observers and a data recorder. During the survey, the pilot recorded the angle of drift away from the flight path. For each pod sighting the observer measured the vertical and horizontal angles from the plane, as well as the GPS location of the plane, and the pod size and composition (number of adults and calves, determined based on size). At the beginning of each flight the devices were calibrated to ±1sec accuracy. Sea state, glare, wind speed, and visibility were also recorded throughout the survey, to monitor changes in sighting conditions. The position of each whale pod was then calculated following the method in Salgado-Kent *et al* [29].

**Table 1:** A summary of the aerial surveys carried out during the 2004 and 2005 whale season (Aug – Nov) in Exmouth Gulf.

Aerial Survey				Distance Sampling			
Flight	Date	Whales	Calves	Detection Function	Abundance	lower CI	upper CI
1	7^th^ Oct 04	135	29	HN (Obs)	409	232	598
2	14^th^ Oct 04	97	16	HZ	144	73	250
3	26^th^ Oct 04	62	12	HZ	71	30	116
4	2^nd^ Nov 04	26	6	HN	44	22	70
5	7^th^ Aug 05	7	0	HN	*	*	*
6	21^st^ Aug 05	35	2	HN	138	54	235
7	4^th^ Sep 05	79	4	HN	248	127	411
8	10^th^ Sep 05	41	3	HN	84	43	126
9	25^th^ Sep 05	126	17	HN (Pod)	459	250	816
10	15^th^ Oct 05	95	13	HN	279	167	413

In the seven other surveys, flown on 18^th^ Feb, 7^th^ Mar, 3^rd^ Apr, 26^th^ Apr, 22^nd^ May, 12^th^ Jun, and 12^th^ Jul 2005, no whales were observed.

The selected detection function for the survey was either a half-normal (HN) or hazard (HZ) function, with two surveys needing an additional covariate of either observer (Obs) or pod size (Pod). The abundance was estimated using this detection function and the 95% lower and upper confidence intervals (CI) were calculated using a bootstrap.

* The abundance was not calculated for flight 5, as the sample size was too small (<20) to obtain reliable results from distance sampling.

### Abundance

Population transect surveys are subject to availability and perception biases, whereby animals could be missed if they were not available to be seen, or they were available but not seen by the observer [34]. Therefore, distance-sampling was used to reduce any errors caused by perception bias and provide more accurate estimates of abundance [32]. This method models the probability of detection of an animal group as a function of the perpendicular distance from the transect. The probability detection function can also take into account the variation in sighting conditions, by introducing covariates such as observer and sea state. Once a detection function has been fit, it is used to estimate the actual number of animals in the survey area, including those likely to have been missed by the observer (the perception bias).

The sightings data were right-truncated at 5 km from the transect line, removing 5% of the data, following the general ‘rule of thumb’ to remove extreme values prior to fitting detection functions [32]. In aerial surveys, it is also difficult to make observations on the transect line as it lies directly beneath the plane. However, the method of fitting a detection function assumes that all animals at the surface (available to be seen) on the transect line were observed. To account for the discrepancy, a standard left-truncation at 0.1 km was set to obtain a better detection function fit, however this did not result in any loss of data as no observations were made within this distance. Distance 6.0 [35] was used to fit different detection function models (half-normal and hazard-rate) for each flight, taking into account covariates that may affect detection probability such as observer, sea state, pod size, and day of flight. Model selection for each flight was based on the Akaike’s Information Criterion (AIC), Q-Q plots, and the Kolmogorov-Smirnov and Cramer-von Mises goodness-of-fit tests. If two or more models were too similar to make a selection based on the above criteria, the parsimonious model was selected. The abundance of whales in Exmouth Gulf for each flight was then estimated in Distance 6.0 using the best probability detection function, and 95% confidence intervals were obtained using a bootstrap.

Availability bias was not accounted for in this analysis, and therefore the model will underestimate abundance. However, we believe this difference to be small as the Gulf is relatively shallow, and resting whales tend to display passive behaviours such as surface lying or surface travelling [36]. Therefore pods are more likely to be at the surface and available to be seen from aerial surveys.

Pods with calves have previously been demonstrated to lag in the migration [37], [38]. By looking at the seasonal variation in calves in the Gulf and comparing this to the total abundance of all whales using the Gulf, we can determine if a lag also exists in resting areas. If there were no lag, this would indicate that mainly mothers with calves are likely to be using resting areas. If a lag does exist, then comparing the length of this lag with those found by Dawbin [38] in the main migration pattern will indicate which groups are using the Gulf. The change in the number of pods with calves over times was plotted to test this prediction for this population of humpbacks. As many of the flights contained a small sample size of pods with calves (<20), distance sampling was not used in this analysis.

### Abundance-Occupancy Relationship

The abundance-occupancy relationship (AOR) describes the relationship between the abundance of a species and the size of their ranges within a region, and reflects the pattern of abundance covarying with the total area occupied [39], [40]. As AOR is usually evaluated across many sites within a region, abundance is calculated as the mean density across all occupied patches, and the occupancy as the sum area of all the occupied patches [40]. In the basic AOR pattern, density remains constant while the occupied area increases, meaning that abundance increases in proportion to the area. However, for most species the AOR is positive; as the density increases so does the occupied area [40]–[44]. In these cases, the population size is increasing at a greater rate than would be expected simply by a range expansion. Alternatively, the AOR pattern may reflect increases in density while the occupied area remains constant. In this case, population abundance increases but the range size stays the same. Understanding the AOR relationship has important implications for conservation; if there is a positive AOR then any reduction in habitat will result in a greater loss in individuals proportional to the AOR [45]. To investigate the AOR for humpback whales in Exmouth Gulf, the occupancy area for each flight was estimated by a convex hull analysis [46], [47], which calculates the minimum area occupied by the population by fitting the smallest polygon possible that encompasses all the humpback whale sightings. The abundance was then estimated by calculating the density of humpback whales within the convex hull area (CHA).

### Factors Affecting Pod Density

To calculate the carrying capacity of pods in Exmouth Gulf, it is important to first determine what factors may influence their spatial organization and nearest neighbour distances. The two factors we investigated here were pod size and pod composition, where a pod is defined as a group of one or more animals. During the breeding season, humpback whales are usually found in pods of 2–3 animals, however pod size can range from 1 to 20 animals [48]. Pod size could affect spacing behaviour in that, for example, larger pods may prefer more space. The type of animals present in a pod may also influence their nearest neighbour distances regardless of pod size. For example, during the breeding season mother and calf pairs receive the attention of adult males who are looking to compete for and mate with the now receptive female [49], which may alter the spacing of animals around pods with a calf.

To investigate the effect of pod size, the nearest neighbour distance for each pod in each flight was estimated. The pods from all the flights were then grouped together based upon pod size, ranging from 1 to 8 animals. As there were less than three observations for pods containing 5 or more animals, these groups were excluded from the analysis. The nearest neighbour distances in each of the remaining four group types were distributed non-normally (Kolmogorov-Smirnov test, p<0.05 for all groups). Therefore, the median nearest neighbour of each group size was compared using a Kruskal-Wallis test.

Pod composition was defined as those with calf present (wCP) and those without (nCP), due to the limitation of aerial surveys to specify composition in more detail, such as singing males. The nearest neighbour distances for each pod in the two categories were also distributed non-normally (Kolmogorov-Smirnov test, p<0.05 for both groups), and thus the spacing around the different pod types was tested by comparing the median nearest neighbours using a Kruskal-Wallis test.

### Spacing Behaviour

**Figure 2:**
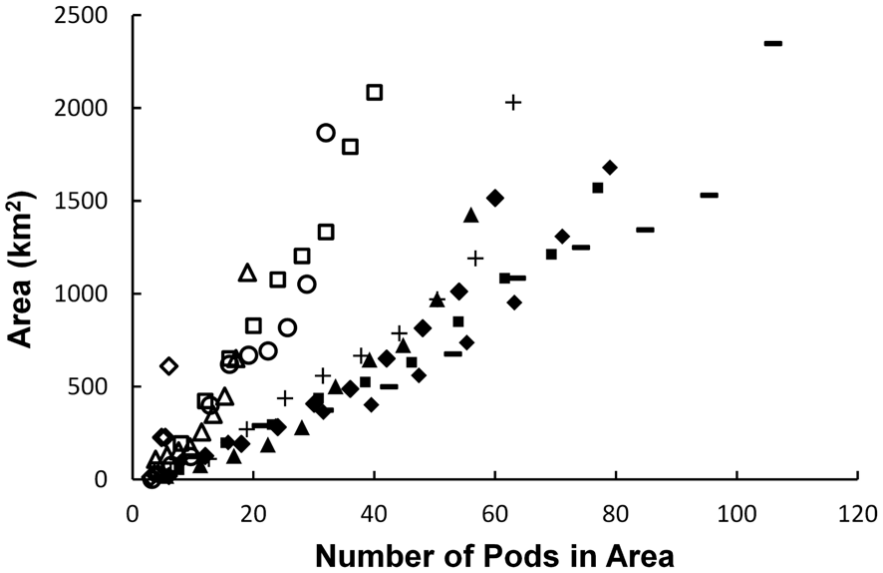
The area occupied by an increasing number of pods nearest to the centre of aggregation. The minimum polygon area (convex hull area) around whale pods was repeatedly calculated for each survey flight to include increasing number of pods closest to the centre of aggregation, starting at the nearest 10% until all pods were encompassed by the polygon. A scatter plot of these changes in area occupied reveals two patterns in area use; the first group of flights are indicated by open symbols (▵ flight 4, ⋄ flight 5, ○ flight 6, □ flight 8) and the second group by closed symbols (♦ flight 1, ▪ flight 2, ▴ flight 3, +flight 7, – flight 9, ♦ flight 10).

We defined spacing behaviour as the distance maintained between pods under relatively dense conditions. To determine the spacing of individual whale pods, we first needed to see if the distribution in the flights followed the same pattern of space use. The harmonic mean position of pods for each flight data set was estimated, and the CHA was then calculated by including increasing percentages of pods closest to the harmonic mean, starting at 10% and increasing to 100% in increments of 10%. Two distinct trends of space use emerged with increasing number of pods included in the analysis (Fig. 2): flights 4, 5, 6, and 8 used more space for fewer numbers of pods due to the low number of whales recorded on those days, due to being at the beginning and the end of the season (Table 1) while flights 1, 2, 3, 7, 9 and 10 occupied less area per number of pods. As we wanted to calculate the distance maintained in relatively high density conditions, flights with low densities (4, 5, 6, 8) were excluded from further analysis.

Two distinct methods were used to calculate pod spacing to assess consistency of the estimates. The first method used the nearest neighbour distances between pods, whereas the second investigated the number of whales within a given radius of a pod.

#### Method 1

**Figure 3:**
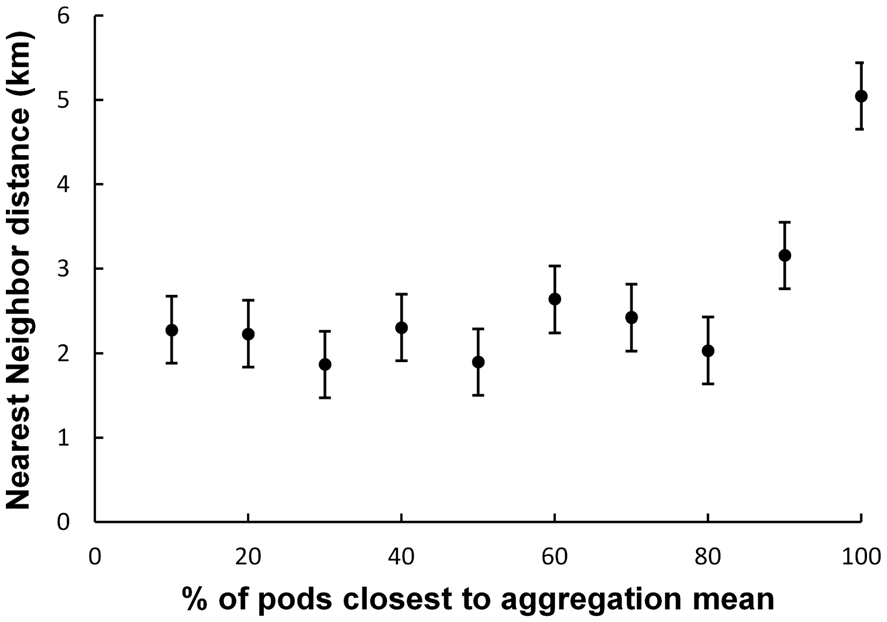
A comparison of nearest neighbour distances with proximity to the centre of aggregation. The nearest neighbour distance (mean of flights ± standard error) of groups of pods based on how close they are to the centre of aggregation, i.e. the 10% mark contains the closest 10% pods to the mean, the 20% mark contains the closest 10–20%, and so on up to the 90–100% group. The only group with a significantly different nearest neighbour distance was the 90–100%, which was much higher than the rest.

For each pod in each flight, the distance to the nearest neighbour pod was calculated. A nearest neighbour analysis [50] was conducted for each flight, which calculates the ratio (Rn) between the observed mean nearest neighbour distance (NND) and the expected mean NND given a random distribution. Randomly distributed animals will give an Rn value of 1, clustered animals will have a value less than 1, and uniformly spaced animals will have a value greater than 1. This analysis indicated that pods in Exmouth Gulf were not uniformly spaced, but had a tendency to cluster (Rn = 0.8; mean across 6 flights). As such, the nearest neighbour distance will vary depending on the distance between the pod and the centre of aggregation. We thus grouped pods based on how close they were to the centre of aggregation because we needed to study the pod arrangement when humpbacks were in relatively high density conditions, each group included 10% of the pods; the 0–10% group contained the 10% of pods closest to the harmonic mean, the 10–20% group was the next closest 10% to the mean, and so on. We used a one-way analysis of variance (ANOVA) to compare the mean nearest neighbour distances for each percentage category. The mean nearest neighbour distances in the 90–100% group were significantly higher than the rest of the groups (p<0.05; Tukey-Kramer. Fig. 3) and so were excluded from this analysis. The nearest neighbour distances of the remaining 90% of the pods were also non-normal (Kolmogorov-Smirnov test, p<0.05), so the overall distance maintained between pods was calculated as the median nearest neighbour distance. As there will be individual variability in pods, variation in this distance was estimated by calculating the median absolute deviation (MAD).

#### Method 2

For each flight, sequential circular boundaries at a radial distance of 0.001 km were drawn around every pod from a minimum radius of 0.001 km to a radius where all the pods had another pod present in the boundary. The proportions of the pods that had at least one pod present within these radii were then calculated. This approach produced a cumulative density of the proportion of pods that had other pods present within a radius of increasing length for each flight. A curve was then fitted to the data using the least squares method [51] and an exponential model. Here, the distance maintained between pods was estimated to be at the 50% mark, analogous to the use of LD50 curves in toxicology [52] and size at maturity curves in fisheries [53]. At this point, half the pods have no other pods within the boundary and half of the pods have at least one other pod within the boundary, providing an estimate of pod spacing for each flight. As a preliminary regression analysis indicated no season trend in the radii (p>0.05), the overall population pod spacing estimate was taken as the mean radius of the six flights, and the error range as the lowest and highest radius over the flights.

### Carrying Capacity

Assuming that all pods maintain an area of space, the maximum number of pods able to fit in Exmouth Gulf at any one time can be estimated as the highest density of pods allowing for distance between pods to be maintained within the area utilized by the population. The distances from the two above methods were used as a radius to determine a circular boundary of space around a pod. The maximum area used by the population of humpback whales was taken to be the CHA around all recorded pods over all the flights. The pods, plus their circular space, were arranged in a lattice formation, the densest concentration of circles on a single plane [54], within the CHA while allowing the circles to overlap to the length of the radius so that the nearest neighbour to a pod was no closer than the spacing radius. This maximum number of pods for each of the methods was then multiplied by the average pod size to obtain two estimates of the carrying capacity for Exmouth Gulf. The error range for method 1 was calculated using the median absolute deviation [55] while the error range for method two was estimated by calculating the carrying capacity from the maximum and minimum 50% radii of the six cumulative density curves.

## Results

**Figure 4:**
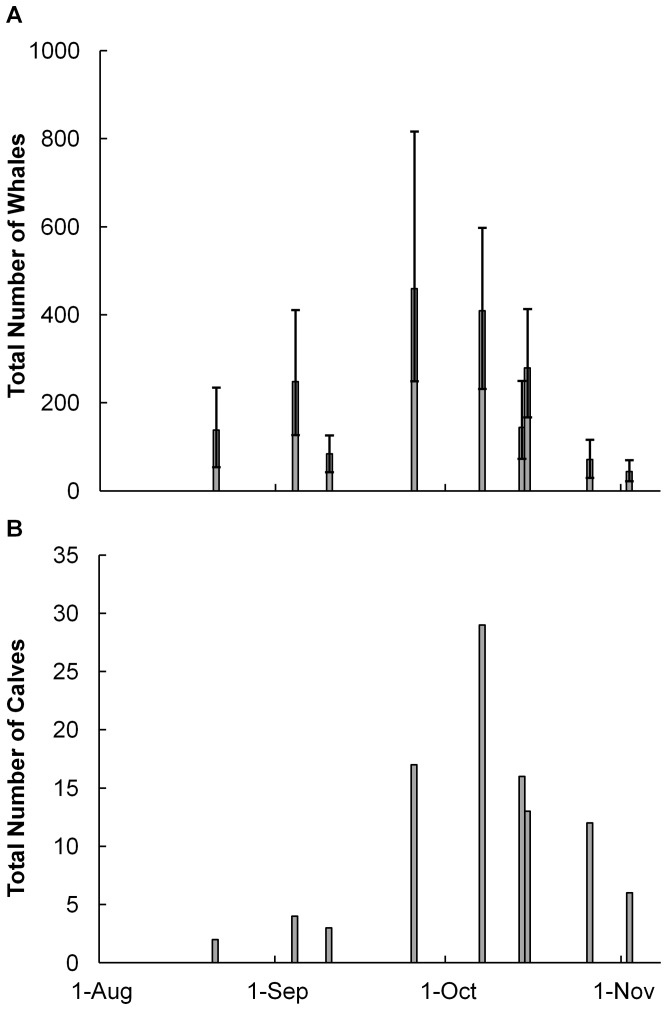
The temporal changes in number of A) total whales, and B) calves, in Exmouth Gulf. A) For each flight, the total number of whales resident in Exmouth Gulf was estimated using distance sampling. The error bars mark the 95% confidence interval, calculated using a bootstrap in Distance 6.0. There is clear temporal pulse of whales in the Gulf, with the peak occupancy towards the end of September. B) The total number of calves observed during each survey flight also displays a temporal pulse to occupancy, but the peak here is slightly later in the first week of October.

A total of 703 individual whales were sighted in the Exmouth Gulf region during the 2004–2005 aerial surveys (Table 1). The abundance estimates for each flight, using distance sampling, showed a maximum of 459 whales within the Gulf at any one time, and a total of 1270 whales over the entire period (CI 670–2080), assuming a maximum two week residency period for each whale (KCS Jenner, estimated from photo ID re-sights; each whale is represented only once in the total estimate). The abundance of whales in the Gulf clearly changed over time (Fig. 4a), with whales beginning to enter the Gulf from the north around the first week of August, peaking at the end September, before departing until the start of November. The number of calves within the Gulf follows a similar temporal pattern, but peaks about a week or two after the main migration (Fig. 4b), in early October.

**Figure 5:**
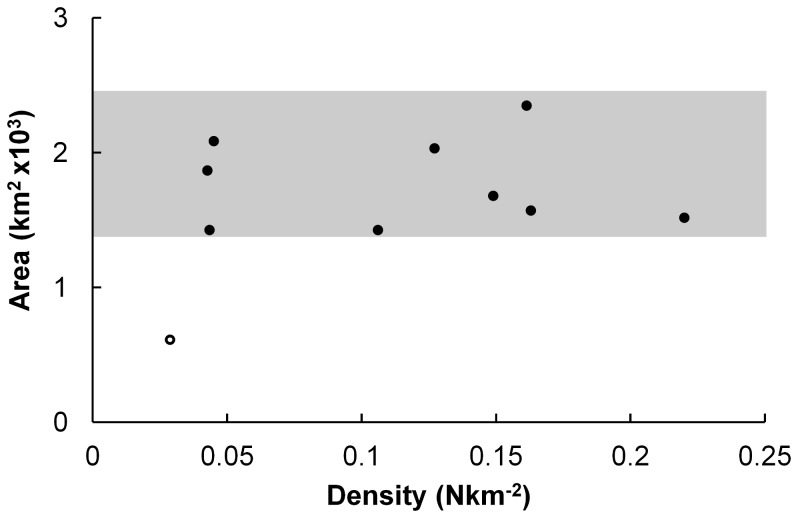
The relationship between density and occupancy of whales in Exmouth Gulf. The total area occupied was calculated as the convex hull area for each flight, and the density as number of whales per km^2^ in this area. The pattern emerging is that of a constant area used with increasing density, as highlighted by the grey shaded area. One survey (flight 5), marked as an open circle, is an outlier to this pattern.

Whales in Exmouth Gulf follow an abundance-occupancy relationship whereby the area occupied remains relatively constant as abundance increases (Fig. 5; shaded area), and consequently density is increasing with abundance. The area of the first value (marked as an open circle) is less than half that of the other areas. This may be an anomaly in observation, or it may indicate that a constant abundance-occupancy relationship exists only above a threshold of at least 0.04 whales per km^2^.

**Figure 6:**
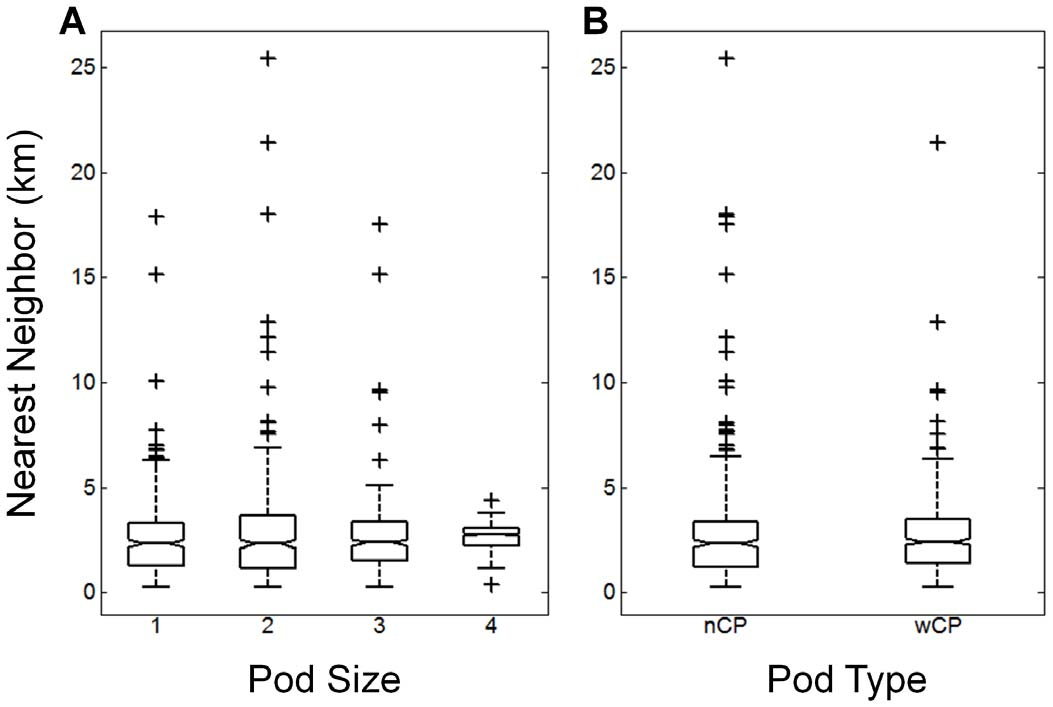
Boxplots comparing median nearest neighbour of A) the pod size and B) the pod type. Neither pod size or type showed significant difference in median nearest neighbour distances (Kruskall-Wallis test: pod size p = 0.80, pod composition p = 0.58). For pod type, ‘wCP’ are pods with calves present and ‘nCP’ are pods with no calves observed.

Estimating the carrying capacity of whales in Exmouth Gulf requires an understanding of how pods spatially organize themselves within the Gulf, which may be influenced by pod characteristics. However, the two characteristics we investigated here, pod size and composition, had no effect on the median nearest neighbour distance of the pods (Kruskall-Wallis test: pod size p = 0.80, pod composition p = 0.58; Fig. 6). Therefore, these variables were not incorporated in analyses to calculate carrying capacity.

**Figure 7:**
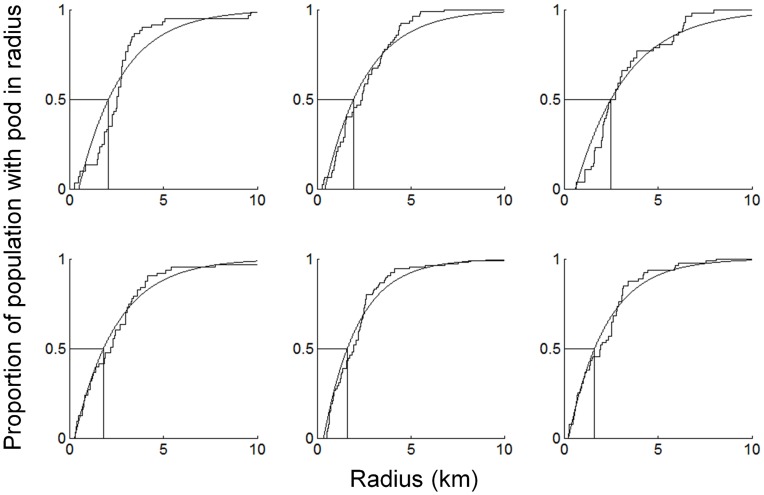
The proportion of the population with a pod within a fixed radius, and increasing radii. Cumulative density plots of the proportion of population that have at least one pod within a specified radius, at increasing radii, for A) flight 1, B) flight 2, C) flight 3, D) flight 7, E) flight 9, and F) flight 10. Each plot was fit with an exponential curve using the least squares method, and the radius at which half the population have a pod within this radius was calculated from the curves. Theses radii are A) 2.09 km, B) 1.95 km, C) 2.50 km, D) 1.82 km, E) 1.61 km, and F) 1.62 km.

The first method for estimating the spacing between pods, using the median NND of 90% of the population, generated a radius of 2.16 km (MAD ±0.94 km). In the second method, the saturation curve fit to an exponential model (Fig. 7) estimated the mean distance within which half the population had a pod as 1.93 km (lowest 1.62 km; highest 2.50 km). Fitting the pods into the maximum CHA area of 2742 km^2^ in a lattice formation yielded maximum pod estimates of 698 (method 1; range 345–2160) and 872 (method 2: range 523–1242). Given an average observed pod size of 1.7 whales, this equates to carrying capacity estimates of 1187 and 1482 whales, respectively, and density estimates of 0.43 and 0.48 whales km^−2^ (Table 2). The two distinct approaches to estimating the distance maintained between pods were within 0.1 km of each other, translating into a difference in total carrying capacity of approximately 175 pods or 295 whales.

**Table 2:** The carrying capacity estimates for method 1 (median nearest neighbour) and method 2 (50% of population with a pod within this radius).

Method	1	2
Radius (km)	2.16	1.93
CC Pods	698	872
Mean pod size	1.7	1.7
CC Whales	1187	1482
Density (whales km^−2^)	0.43	0.54

CC: Carrying capacity.

The carrying capacity of pods was calculated by fitting the maximum number of pods, including their radius distance, into the convex hull area encompassing the entire population. The carrying capacity of whales is the number of pods multiplied by the mean pod size.

## Discussion

Our premise is that in limited-space conditions the carrying capacity of an area for resting humpback whales is linked to the space requirement of the animals that occupy it, rather than more typically encountered pressures such as competition for food, and predator avoidance. Our results suggest that pods do maintain a distance from each other under relatively high-density conditions, demonstrating that space itself is a resource for these animals and that this space can be determined. We then used this spacing distance to calculate the theoretical carrying capacity of a humpback whale resting area. The implications of having a capacity limit, under the currently increasing population of WA humpback whales, can only be assessed once the current use of Exmouth Gulf is understood. Therefore, we also investigated how the humpback whale population presently uses Exmouth Gulf, both spatially and temporally.

There is a clear temporal pulse to peak whale occupancy of Exmouth Gulf, starting from late September to early November, which conforms with the timing of movement of the whale population down the WA coast [31], [37]. This temporal pulse could be caused by an environmental signal that triggers the whales to leave particular areas and continue on their migration, such as a change in temperature or day length, however the influence of environmental cues on the migration of baleen whales is still poorly understood [56]. As adult humpback whales need to complete migration before energy reserves are exhausted, leaving the Gulf may also be triggered by a certain level of depletion in these reserves.

The number of pods containing calves peaks in the Gulf after the main migration, supporting previous observations that mothers with calves follow the main migration [37], [38]. The timing offset between the two peaks in this study was approximately two weeks, which is shorter than that estimated by Chittleborough [37]. However, the Chittleborough [37] study used commercial catch data, which reflect hunting near migratory areas and thus were likely to capture the timing of the entire population of migrating whales, whereas our data were concentrated on a resting area. Therefore, the disparity between peak timings could mean only a portion of the migrating whales are using Exmouth Gulf to rest during the southbound migration; if the vanguard of the migrating population are not using Exmouth Gulf then the peak of whale abundance in the Gulf would appear to be later than if sampling the entire population. The match in timing difference between peaks of mature males and lactating females found by Dawbin [38] indicates that it is these groups of whales which are mostly present in the Gulf, supporting the conclusion that resting areas are particularly important for mothers with calves [57], but also that it is an area where mature males and lactating females are mating. It is important to note that, as distance sampling was not applied to the calf data due to the small sample sizes, the perception bias (the calf is available to be seen, but is not observed) was not corrected for, and thus the total number of calves within the Gulf could not be estimated. To further investigate calf presence in this resting area will require more detailed surveys.

The importance of resting areas to migrating whales is still unknown, however during migration a calf requires sufficient food from its mother to enable it to grow and gain adequate energy reserves to continue migrating towards the Southern Ocean. Mothers are therefore expending their own limited stores to meet both their own energetic requirements and that of the calf. Spending time resting in sheltered embayments, such as Exmouth Gulf, during migration allows calves to increase energy stores more efficiently, as they will be expending less energy when compared to resting and feeding in open ocean conditions, and thus slowing the rate of energy loss. Furthermore, wind speed is known to influence the energetic surface active behaviours of humpback whales, with rising wind speed increasing behaviours like breaching, pectoral fin slapping, and tail slapping [58]. This correlation is linked to a change in communication strategies during periods of higher wind-dependant background noise [58]. Therefore, the flatter the surface conditions, the more the humpback whales can rest. The wind conditions in Exmouth Gulf are typically characterized by diel changes in speed, creating calmer conditions during the day and for several hours wind speed can drop to nothing. During the October period in northern Western Australia, these extended low wind, flat water conditions are unique to Exmouth Gulf. These conditions create the ideal resting environment along the Western Australian coast for whales, particularly mother with calves, at perhaps a critical stage of their migration towards polar waters. In the Australian context, this unique opportunity to boost calf energy reserves mid-migration may increase long term survivability of calves for this population and partly explain the higher population growth rate measured in west Australia’s Stock D versus east Australia’s Stock E [29], [59]. Considering resting areas are predominately used by mother and calf pods, we theorise that these pods are driving the spacing behaviour in this resting area, perhaps due to mothers regulating the social stimulus of the calf. However, further research will be required to determine if this is the driver behind the spacing behaviour.

### Abundance-Occupancy

The abundance-occupancy relationship of humpback whales within the Gulf demonstrates that the total space used within the Gulf remained constant regardless of whale abundance. The one exception to this rule is at the lowest abundance observed suggesting there could be a positive AOR below a particular threshold of whales. However this holds little significance to the overall understanding of space use as for the majority of the time, the Gulf is occupied at abundance levels above this threshold value.

The AOR analysis uses the average density of whales across the total space used by all the whales (the CHA area) over the duration of each flight, and therefore does not capture any information on the arrangement of whales within this area. However, this spatial arrangement is an important consideration as it has the potential to confound the spacing behaviour analysis. Given that the area used remains constant, if pods are spacing evenly throughout the area then the distance maintained between pods will decrease as density within the area increases. Alternatively, if the pods are aggregating within the area, then average maintained distance will remain relatively constant and core area of aggregation will continue to expand within the limits of available resting area. The nearest neighbour analysis indicated a tendency towards aggregative behaviour, suggesting the second spatial arrangement. As outlying pods, the 90–100% furthest away from the centre for aggregation, were removed from the spacing behaviour analysis, the results are not confounded by having a constant AOR.

The constant AOR relationship found for humpback whales in the Gulf is different to other cetacean species investigated [44], which tend to show a positive AOR. This could reflect the difference in the population’s situation at the time of study; for example, the Minke whales (*Balaenoptera acutorostrata*) were analysed while they were foraging [44], whereas the humpback whales in this study were not feeding and so distributions were not driven by food patchiness. The consequence to having a constant AOR is that as more whales enter the Gulf, the Gulf becomes increasingly dense. Given that there is a minimum requirement for space between individual pods, at some point maximum density, and thus carrying capacity, will be reached.

### Implications of Spacing Behaviour

The two approaches we took to calculate spacing between pods arrived at very similar estimates of approximately 2 km between pods. While both estimates are derived from the same data set, the answers are distinct due to fundamental differences in the approaches used in the estimation of spacing distance. The first method concentrated on only the measured distance to the nearest neighbour for each pod, ignoring any other pods in the vicinity, while the second approach took account of all the pods within a given radius, regardless of which was nearest.

Pod size and composition did not affect the distance maintained between pods, and so density of pods within the Gulf will be the same regardless of these factors. There were very few observations of pods containing more than four animals, so an effect on nearest neighbour distance may still exist at higher pod sizes. However, given the few instances of large pod sizes in Exmouth Gulf over the season, this will have little consequence on the overall pod carrying capacity within the Gulf, assuming that a recovering population does not lead to larger pods. Pod composition could also be further disaggregated to investigate difference between, for example, singing males or competitive males. However, these aerial survey derived data did not allow us to distinguish such individuals. Other factors that may influence pod density, but were not able to be investigated here, are pod activity, habitat preference, and competitive exclusion. However, the distance maintained between pods calculated here is representative across the population occupying the Gulf at a point in time, so represents an average over pods in various states of activity and habitat preference. Competitive exclusion is likely to be a factor only once the Gulf approaches maximum density and space to arriving whales becomes unavailable, which appeared not to be the case in the 2004–2005 seasons. Another potentially confounding variable when calculating the spacing distance of pods is movement of and interaction amongst pods. However any extremes in this variation, such as a closer than normal distance between interacting pods, or larger than normal from pods requiring more space, would be accounted for in the analysis by looking at the central tendency in distances, resulting in a representative spacing distance across the population occupying the Gulf at that point in time.

The knowledge that resting humpback whales maintain spacing has implications for their interactions with vessels. Seismic vessels have strict guidelines when operating around and approaching whales, which outline observation, low power, and shutdown zones depending on the distance from the whales [60]. The 2 km spacing of whale pods matches the 2 km low power zone for vessels operating above 160dB [60], however below this source level the low power zone is reduced to 1 km, which could be viewed as an invasion of space for the pod. Tourism vessels also have guidelines when approaching whales [61], with a caution zone of 300 m and a no approach zone of 100 m while fishing vessels have to keep a distance of at least 100 m, all of which are well within the behavioural spacing of humpback pods as calculated here. A specific humpback whale sanctuary established in Camden Sound, the calving grounds for this population [31], has increased this 100 m no approach zone to 500 m for mother and calf pods, however this still falls short of the 2 km distance maintained between pods found in this study. We do not dispute that these regulations are adequate to avoid disturbance to the whales, indeed the population is recovering at near maximum rate [29], [62], however vessels spending too long within the boundary of a pods’ space may end up increasing calf interaction and activity levels, and therefore energy consumption, at a time when net energy levels are intended to be increasing. So while the immediate impact of displacement and/or increased activity may not be apparent, there may be longer term implications to the survivability of the calf mid-migration which is drawing on fixed energy reserves from its mother. We would therefore recommend a precautionary approach to management decisions when considering increasing vessel density in areas likely to contain resting whales.

We calculated the theoretical carrying capacity of Exmouth Gulf to be around 700–850 pods (1200–1500 whales), based on the spacing between pods and the maximum CHA used by the whales. There are moderate errors surrounding the carrying capacity estimates, with ranges calculated as 345–2160 pods and 523–1242 for methods 1 and 2 respectively, but our estimates of overall carrying capacity are comparable. Considering the constant AOR relationship, whereby the population is occupying the same amount of area regardless of the number of whales within the Gulf, there will likely be no change in the total area used by the whales until carrying capacity is reached. The area the whales are currently occupying (2,742 km^2^) encompasses most of the Gulf. In the context of a currently increasing numbers, there is little room within the Gulf for the expansion of whale populations. Therefore, if the response of the population is to expand the resting area, then this expansion will extend outside of the Gulf. Alternatively, the whales will seek other appropriate areas in which to rest along the coast, which is of particular concern given the current coastal developments in the northwest of Australia for extractive industries. It is also important to realize that, as space is the limiting factor for carrying capacity, then any reduction of space within the Gulf available for whales to rest will result either in a reduction to the total carrying capacity of the Gulf or a decrease in spacing between pods. As maximum carrying capacity in the Gulf was not observed in this study, it is difficult to predict the consequence of reaching carrying capacity based on space limitations.

To calculate carrying capacity, this study assumed that space limitation exists in the Gulf. The consistent area occupied by the whales over varying densities suggests that there are physical constraints with respect to the area used by humpback whales, making space a limited resource. To determine whether the approximate 2 km spacing distance found in the 2004–2005 seasons is maintained across years will require additional appropriate aerial surveys such that interannual variability in spacing distance as a function of population size can be evaluated. It may be that as the population off the West Australian coast continues to grow, the average space between pods will decrease to accommodate the increase in whales. However, this will depend on the drivers behind spacing behaviour between resting pods. Here, we used the 2004–2005 season to illustrate the concept that space is a resource for resting whales, and distance maintained between pods can be used to calculate carrying capacity at a given point in time, which has important management applications. This study forms the foundation to further work exploring the spacing behaviour of wide-ranging megafauna, and how this may limit carrying capacity in space-limited areas.

### Conclusion

Our study shows that carrying capacity for humpback whales can be calculated based on their behavioural space requirement under relatively dense conditions regardless of pod size or composition, and that this distance can be consistently estimated using two separate approaches. We estimated the carrying capacity of Exmouth Gulf, a migration resting area, to be approximately 1187–1482 whales. Although there has been considerable research into the spacing of other aggregating animals, such as fish and birds, this study is a new approach to understanding the habitat use of large ocean wildlife when they are not feeding, and how the spacing behaviour can determine a habitat’s carrying capacity. The consequence of a carrying capacity in Exmouth Gulf is that, when exceeded, the resting area may expand in time or space, or the whales will begin to utilize other areas along the coast for resting. Given that the whale population is sharing the coastal waters with human activities, such as mining developments, it will be important to ensure any expansions in resting area habitat use are monitored and that the areas whale populations expand into are disturbance free, in order to promote the continued healthy population growth for this recovering species.
